# Investigation of Ternary Mixtures Containing 1-Ethyl-3-methylimidazolium Bis(trifluoromethanesulfonyl)azanide, Ethylene Carbonate and Lithium Bis(trifluoromethanesulfonyl)azanide

**DOI:** 10.3390/ijms17050670

**Published:** 2016-05-04

**Authors:** Andreas Hofmann, Matthias Migeot, Lukas Arens, Thomas Hanemann

**Affiliations:** 1 Karlsruher Institut für Technologie (KIT), Institut für Angewandte Materialien—Werkstoffkunde (IAM-WK), Hermann-von-Helmholtz-Platz 1, 76344 Eggenstein-Leopoldshafen, Germany; matthiasmigeot@hotmail.de (M.M.); lukas.arens@kit.edu (L.A.); thomas.hanemann@kit.edu (T.H.); 2 Institut für Mikrosystemtechnik, Universität Freiburg, Georges-Köhler-Allee 102, 79110 Freiburg, Germany

**Keywords:** ionic liquid, conductivity, density, viscosity, electrolytes

## Abstract

Temperature-dependent viscosity, conductivity and density data of ternary mixtures containing 1-ethyl-3-methylimidazolium bis(trifluoromethanesulfonyl)azanide (EMIM-TFSA), ethylene carbonate (EC), and lithium bis(trifluoromethanesulfonyl)azanide (Li-TFSA) were determined at atmospheric pressure in the temperature range of 20 to 80 °C. Differential scanning calorimetry (DSC) measurements were performed to characterize phase conditions of the mixtures in a temperature range of −120 to +100 °C. The viscosity data were fitted according to the Vogel-Fulcher-Tammann-Hesse (VFTH) equation and analyzed with the help of the fractional Walden rule. In this study, fundamental physicochemical data about the mixtures are provided and discussed as a basis for structure-property relationship calculations and for potential use of those mixtures as electrolytes for various applications.

## 1. Introduction

Mixtures of ionic liquids and organic carbonates are often mentioned as potential electrolyte solvents for use in various electrochemical approaches, e.g., supercaps, alkali metal ion–based batteries or solar cells [1,2,3,4,5,6,7,8,9,10,11,12,13,14]. However, the use of those electrolytes often requires the addition of conducting salts to increase the number of available ions. If the electrolytes are discussed in the context of lithium-ion batteries, lithium bis(trifluoromethanesulfonyl)azanide (Li-TFSA), also known as lithium bis(trifluoromethanesulfonyl)imide (Li-TFSI, LiTFSI) [15], is one of the commonly used salts based on its superior temperature stability, electrochemical stability and lithium-coordinating ability compared to LiPF_6_, LiClO_4_, LiBF_4_ or lithium bis(fluorosulfonyl)azanide (Li-FSA) [2,11,16]. Nevertheless, very few systematic studies about physicochemical properties (viscosity, conductivity, density, phase transitions) of ternary mixtures composed of ionic liquids, organic solvents and lithium conducting salts have been done so far. Mostly selected ratios of solvents and conduction salts are described only [4,10,11,17]. More often, systematic studies of binary conducting salt/solvent mixtures are described including ionic liquids as solvents. The molecular structure of Li-TFSA in organic solvents or ionic liquids is studied by several techniques including Raman spectroscopy and nuclear magnetic resonance (NMR) spectroscopy [18,19,20,21,22,23,24,25,26]. However, for an understanding of chemical and physicochemical behaviors of ternary mixtures and for studying the complex interplay between multiple ions and organic molecules, systematic studies with reliable experimental proof are essential. Such experimental data provide the basis for theoretical calculations [27,28] and the evaluation of applications. The compounds 1-ethyl-3-methylimidazolium bis(trifluoromethanesulfonyl)azanide (EMIM-TFSA), ethylene carbonate (EC) and Li-TFSA are chosen based on their prospective properties for electrochemical applications. In this study, ternary mixtures of EMIM-TFSA, EC and Li-TFSA are presented and discussed in detail in terms of fundamental rheological, conductivity, density and DSC data.

## 2. Results and Discussion

**Figure 1:**
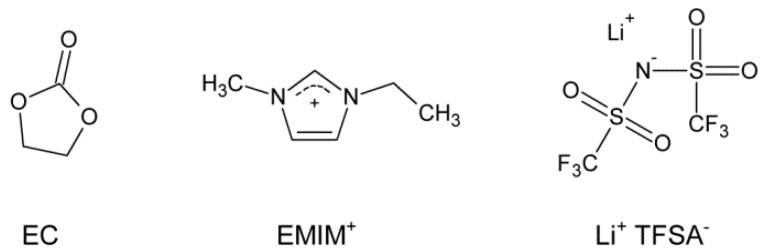
Chemical structures of ethylene carbonate (EC), 1-ethyl-3-methylimidazolium (EMIM) and lithium bis(trifluoromethanesulfonyl)azanide (Li-TFSA).

In this study, the ternary mixture of EMIM-TFSA/EC/Li-TFSA is investigated with respect to density, viscosity, conductivity and phase transitions. The chemical structures of the compounds are displayed in Figure 1. All ratios are listed as wt/wt-ratios and the amount of Li-TFSA is given in mol Li-TFSA related to 1 kg mixture (total mass of all mixture components, specified as mol·kg^−1^). Details about the binary mixture EMIM-TFSA/EC are described elsewhere [29]. Detailed data of the measurements and theoretical fits are provided in the Supplementary Materials as mentioned in the following sections. All ternary mixtures are completely soluble at room temperature. However, heating of the mixtures accelerates the dissolving process, especially for mixtures with high concentrations of Li-TFSA.

**Figure 2:**
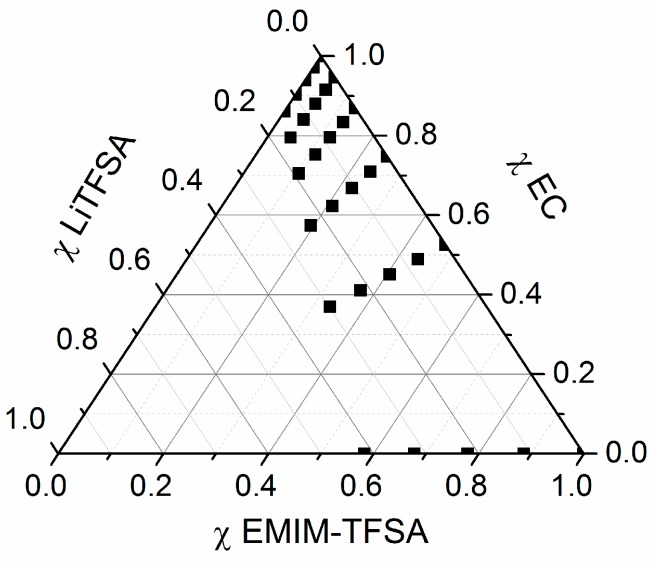
Visualization of the mixtures in ternary mole fraction diagram.

The concentration of Li-TFSA in the mixtures is chosen to be between 0 and 1.2 mol·kg^−1^. These concentration ranges are selected by applications of diluted salt solutions. Principally, the conducting salt concentration can be increased further, especially in the case of high EC content in the binary solvent mixture. It should be noted that *c*(Li-TFSA) = 1.2 mol·kg^−1^ corresponds to a concentration of 1.82–1.95 mol·dm^−3^ on dependence of the EMIM-TFSA/EC mixture (*T* = 20 °C). An overview about the mixtures is visualized in a ternary mole fraction plot in Figure 2. An overview about the measurement data at *T* = 20 °C is provided in Table 1.

**Figure 3:**
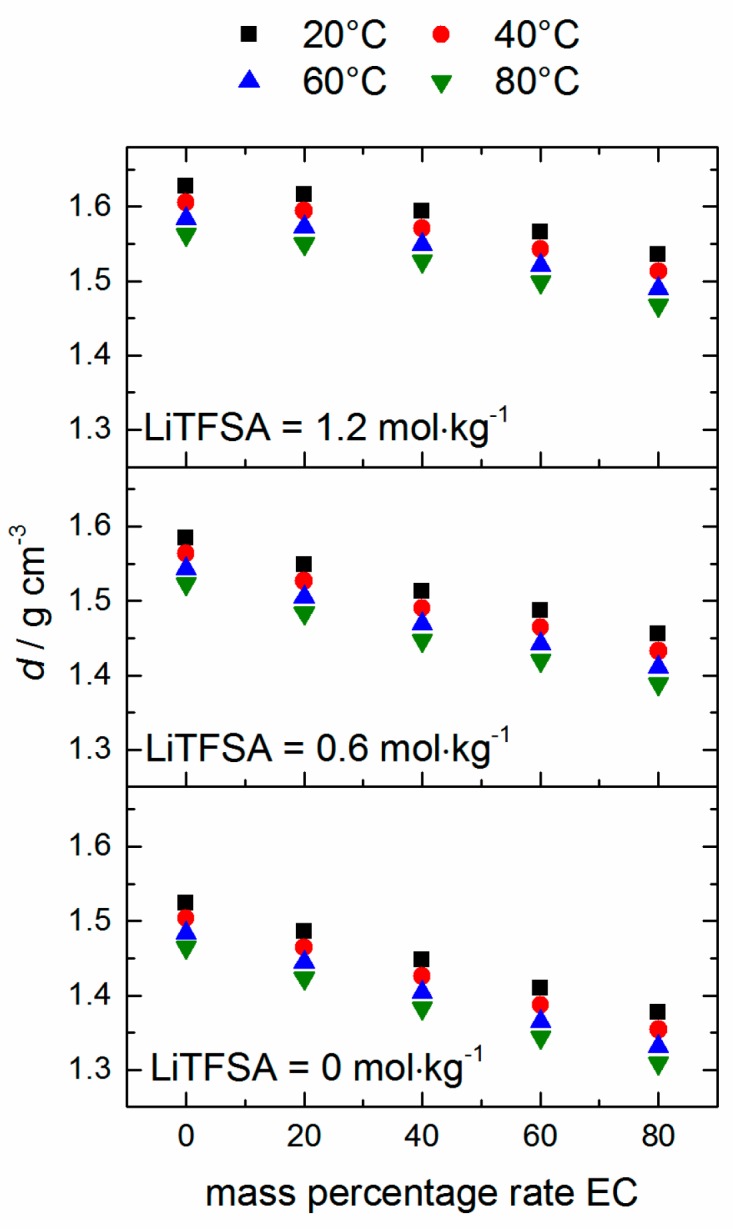
Density values of selected mixtures at *T* = (20–80) °C.

Density values of the mixtures are measured with Li-TFSA concentrations of 0, 0.6 and 1.2 mol·kg^−1^. The results of the measurements are depicted in Figure 3 and the values are listed in Table S1. Linear fittings of the temperature-dependent density according to mass percentage rate EC provide slopes of (−1.86 ± 0.04) × 10^−3^ (0 mol·kg^−1^ Li-TFSA), (−1.58 ± 0.06) × 10^−3^ (0.6 mol·kg^−1^ Li-TFSA) and (−1.22 ± 0.07) × 10^−3^ (1.2 mol·kg^−1^ Li-TFSA), which means that the dependency of the density value on EC mass content becomes smaller with the increasing Li-TFSA content. The error of the slope was determined by applying Gaussian error propagation and the fitting data are listed in Table S2. For all mixtures, the deviation of the real density from the linear fitting is <0.5%, but a general dependency could not be observed (Figure S1a). A polynomial fitting (second order) of the density data led to slight improvement of fitting characteristics (*R*^2^ value, compare Table S2b *vs.* Table S2a), although a *R*^2^ value of <0.99 is received for 1.2 mol·kg^−1^ Li-TFSA mixtures as well.

**Figure 4:**
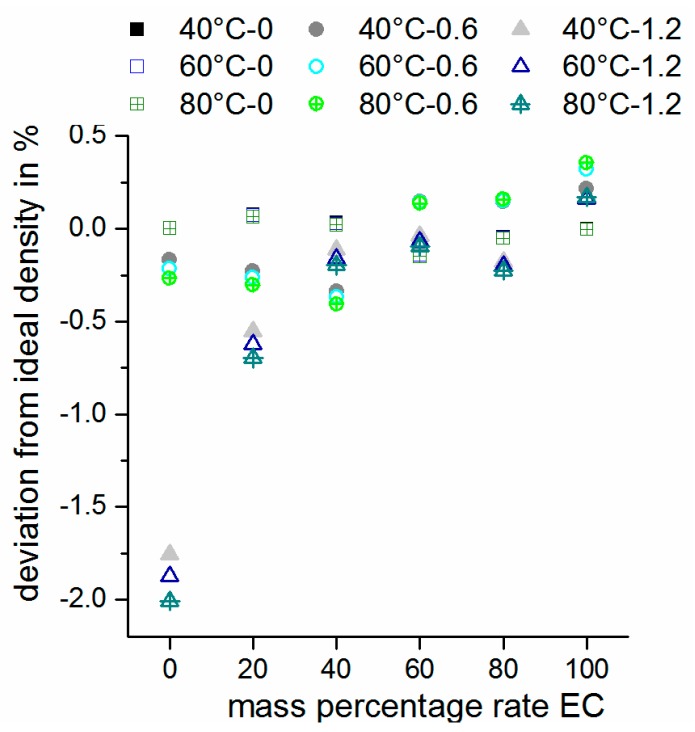
Density deviation from ideal behavior with Li-TFSA concentrations of 0 mol·kg^−1^ (square), 0.6 mol·kg^−1^ (spherical) and 1.2 mol·kg^−1^ (triangle). Mass percentage rate EC is referred to EMIM-TFSA/EC mixture ratio.

The molar excess volume $V_{E}^{m}$ is defined as the difference of the ideal molar volume which is the sum of all individual molar volumes of the components in the mixture, namely $V_{EC}^{m}+V_{EMIM-TFSA}^{m}+V_{Li-TFSA}^{m}$, from the real molar volume $V^{m}$. Usually, such excess volumes are discussed in terms of liquid mixtures because of accessible density values and less pronounced ionic species. The more the attractive or repulsive interactions of the individual molecules in the mixture, the greater the absolute deviation from the ideal volume will be. Therefore, ionic species and salts will form largely solvated ionic interactions and influence the real volume significantly. Kubota *et al.* [30] measured the density of Li-TFSA at high temperatures (260–300 °C) and postulated a linear temperature dependency (*T* in Kelvin) of *d*(*T*) = *A* − *B × T* with *A* = 2.27 g∙cm^−3^ and *B* = 10 × 10^−4^ g∙cm^−3^∙K^−1^. By using this expression, Li-TFSA density values were calculated at temperatures of 40, 60 and 80 °C. The ideal molar volume ($V_{id}^{m}$) and ideal density ($d_{id}$) values of the mixture (using the calculated Li-TFSA density values from Reference [30]) were calculated assuming non-interacting components by applying Equation (1). The deviation of the real density of the mixtures is depicted in Figure 4. Based on these assumptions, molar excess volumes ($V_{E}^{m}$) were calculated as well (see Figure S1b).

(1) $$d_{id}=\;\frac{m}{\frac{m_{EC}}{d_{EC}}+\;\frac{m_{EMIM-TFSA}}{d_{EMIM-TFSA}}+\frac{m_{Li-TFSA}}{d_{Li-TFSA}}}$$

It is remarkable that even at *c*(Li-TFSA) = 0.6 mol·kg^−1^, the deviation of the real density from the ideal density is <0.5% for all solvent ratios. Larger effects are observed only at *c*(Li-TFSA) = 1.2 mol·kg^−1^. The molar excess volume strongly increases at high Li-TFSA concentrations in case of high EMIM-TFSA content (Figure S1b). This can be ascribed to the repulsive interactions in pure ionic systems. Based on its very small size and correspondingly highly charged nature, Li^+^ ions in TFSA^−^-containing ionic liquids (ILs) can be complexed by several TFSA^−^ anions, mentioned as [Li(TFSA)_n_]^1−n^ [31]. However, these cluster-like structures can be surrounded by EMIM^+^ ions which are much larger and less polarized. Presumably, these [Li(TFSA)_n_(EMIM)_m_]-structures, which are polarized by positive charges in the outer sphere, repel each other and lead to an increase in excess volume. When EC is added to such a mixture, small EC molecules can easily form intermolecular interactions with these ionic structures and reduce the repulsive interactions between equally charged ions (EMIM-EMIM interactions). The molar excess volume increases with the enhanced Li-TFSA concentration. In this study, the following trends were received: −0.2 < $V_{E}^{m}$ < 0.2 (EMIM-TFSA/EC); −0.3 < $V_{E}^{m}$ < 0.7 (EMIM-TFSA/EC/0.6 mol·kg^−1^ LiTFSA); −0.2 < $V_{E}^{m}$ < 4.5 (EMIM-TFSA/EC/1.2 mol·kg^−1^ LiTFSA). Based on pure EMIM-TFSA/LiTFSA binary mixtures, it can be observed that the thermal expansivity (indicated by $\left|V_{E}^{m}\right|$) increases with the increasing temperature. This trend is observed less markedly for all samples as well, even at higher EC concentrations. This result suggests that the interactions between the ionic cluster-like [Li(TFSA)_n_(EMIM)_m_] structures become more pronounced when the temperature is raised. It demonstrates that these cluster structures are quite stable and higher temperatures are necessary to break and loosen these aggregates. It is also possible that enhanced temperatures facilitate the formation of these complexes based on increased ion mobility in the mixture.

**Figure 5:**
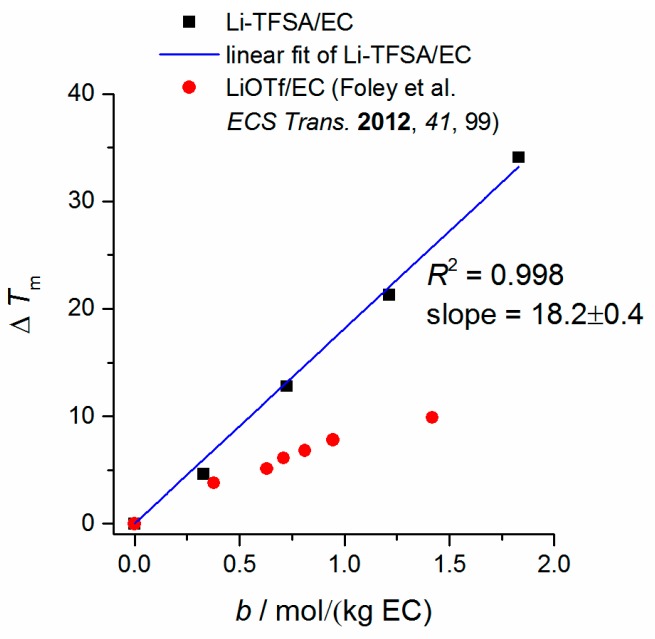
Relative depression of the freezing point *vs.* molality of EC/Li-TFSA compared to results of EC/LiOTf binary mixture from Reference [37]. The result of the linear fitting is depicted as blue line (Δ*T*_m_ = 0 at *b* = 0 was fixed).

DSC measurements of the mixtures are performed to evaluate melting conditions *T*_m_ and glass transition temperatures *T*_g_. Corresponding data of these properties are provided in Table 1. DSC curves are displayed in Figure S2. Trends can be observed in mixtures of ≤60 wt % EC (solvent wt-ratio). In this case, the glass transition temperature *T*_g_ increases when the Li-TFSA concentration is increased as well. This can be ascribed to an increase of the attractive interactions in higher-concentrated Li-TFSA mixtures between the ionic species. The data follow the trend of binary Li-TFSA/IL mixtures, e.g., Li-TFSA/EMIM-TFSA [32], Li-TFSA/1,10-bis(2,3-dimethylimidazolium) decane-TFSA [33], Li-TFSA/dialkylpyrrolidinium-TFSA [34] and others [35,36], where an increase of *T*_g_ by increasing the Li-TFSA mole fraction is mentioned. In addition, a glass transition could be proved at low Li-TFSA concentrations, which had not been visible in the literature before [32]. However, at higher EC concentrations, such a trend (increase of *T*_g_) is no longer observed. All EMIM-TFSA/EC binary mixtures exhibit an endothermic peak (eutectic mixture) at *T* = −27.0 °C which is independent of the mixture composition and which is not present in pure compounds as well as in Li-TFSA mixtures [29]. We assume that this transition results in a molecular picture from an EMIM-EC interaction which enables the formation of small clusters and which disintegrates when Li-TFSA is added. When the Li-TFSA concentration is increased, a decrease in melting temperature *T*_m_ is observed independently from the solvent ratio which is interesting for a possible use of these mixtures at lower temperatures (when pure EC or pure EMIM-TFSA is already in a solid state). It can be ascribed to the general observation of freezing point depression when a solute is dissolved in a solvent. A depression of the freezing point is also described in EC/lithium triflate (LiOTf) binary mixtures [37]. However, a less strong temperature decrease of *T*_m_ is observed for the EC/LiOTf mixture (Δ*T*_m_ = 10 °C; *c*(LiOTf) = (0–1.16) mol·kg^−1^; 5 K·min^−1^) referred to EC/Li-TFSA (Δ*T*_m_ = 34 °C; *c*(LiTFSI) = (0–1.2) mol·kg^−1^; 10 K·min^−1^). Therefore, the cryoscopic constant, *K*_f_, which relates molality *b* to freezing point depression Δ*T*_m_ (Equation (2)), is larger and can be quantified as *K*_f_ = 18.2 ± 0.4 for the EC/Li-TFSA (solvent/salt) binary mixture (*K*_f_ = 7.8 for EC/LiOTf at *c* = (0–1.16) mol·kg^−1^; *R*^2^ = 0.988; calculated from data taken from Reference [37]). The fitting and comparison to literature [37] is depicted in Figure 5.

(2) $$\Delta T=K_{f}b$$

**Figure 6:**
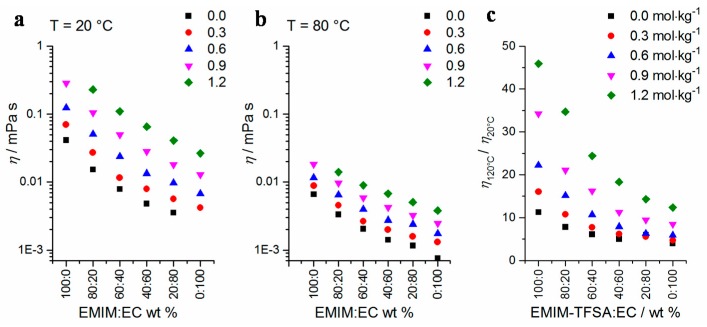
Viscosity of the ternary mixtures at two selected temperatures ((**a**) 20 °C; (**b**) 80 °C); In (**c**), the temperature dependency is shown by the quotient of *η*_120°C_ and *η*_20°C_.

**Figure 7:**
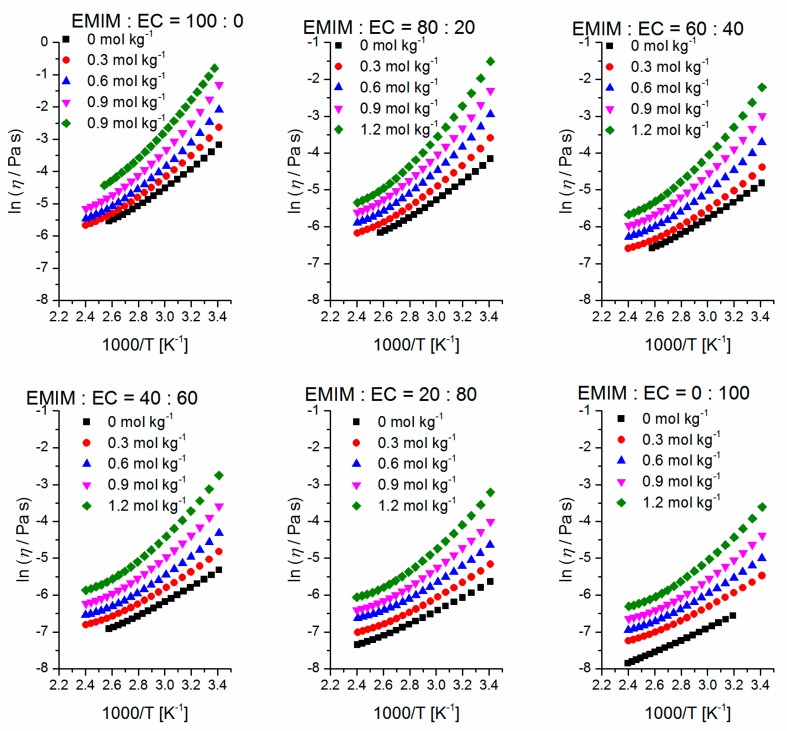
Temperature-dependent Arrhenius plots of the mixtures’ viscosity data in the temperature range of 20–120 °C. The measurements are performed on a 40 mm/1° cone with a heating rate of 3 K·min^−1^ at shear rate of $\dot{\gamma }$ = 100 s^−1^. The concentration of Li-TFSA is mentioned between 0 and 1.2 mol·kg^−1^.

In Figure 6, the dynamic viscosity values of the mixtures are depicted at *T* = 20 and 80 °C. Temperature-dependent Arrhenius plots of the mixtures’ viscosity data in the temperature range of 20–120 °C are shown in Figure 7. By increasing the Li-TFSA concentration, the viscosity values of the mixtures increase gradually. At the same time, the viscosity values of the mixtures decrease when the amount of EMIM-TFSA is decreased. The highest value of the viscosity at *T* = 20 °C (*η* = 556 mPa·s) is obtained in the case of pure EMIM-TFSA with a Li-TFSA concentration of 1.2 mol·kg^−1^. A measure of the temperature dependency of the viscosity is the quotient *η*_T1_/η_T2_ at two different temperatures, T_1_ and T_2_ (Figure 6c, *η*_120°C_/*η*_20°C_). A strong correlation is observed at high EMIM-TFSA concentrations, whereas the temperature dependency is smaller at high EC concentrations. The temperature dependency of the viscosity increases significantly at higher Li-TFSA concentrations, especially between 0.9 and 1.2 mol·kg^−1^.

The Vogel-Fulcher-Tammann-Hesse (VFTH) Equation (3) was used to fit the experimental viscosity data and to characterize the molecular motion with respect to the fitting parameters *η*_0_ (limiting viscosity), *B* (fitting parameter) and *T*_0_ (ideal glass transition temperature). The Angell strength parameter *D* describes the “strength” of a liquid with regards to the coordination of a molecule in the liquid phase [38,39] and can be derived from the parameters *B* and *T*_0_. The “*m*” fragility, “steepness index” or “fragility parameter *m*” is related to the Arrhenius activation energy via the slope of the effective activation enthalpy at *T*_g_ and can be expressed in terms of the parameters of the VFTH equation [39,40,41,42]. By assuming a minimum value of *m*_min_ ≈ 16, *m* can be calculated according to *m* = 16 + 589.5·*D*^−1^ [40,42] and can be used as a measure of the fragility of the mixture [43] based on the fragility concept introduced by Angell *et al.* [39,44,45].

(3) $$\eta =\;\eta_{0}\cdot \exp\left(\frac{B}{T-T_{0}}\right)=\eta_{0}\cdot \exp\left(\frac{D\;\times \;T_{0}}{T-T_{0}}\right)$$

The fitting data for temperature-dependent viscosity values of the ternary mixtures are listed in Table S3 (Supplementary Materials). A free fitting without applying additional data points at the glass transition temperature (e.g., 10^10^ or 10^12^ Pa·s [43,44,45]) was performed within good correlations of *R*^2^ ≥ 0.9995. In the fitting procedure, an Angell strength parameter *D* of 2.3 < *D* < 7.1 (fragile for *D* < 30) and a fragility factor m of 99 < *m* < 277 are received. Pure EMIM-TFSA has already been investigated by Schreiner *et al.* [46] where comparable values of *η*_0_ (here: 18.3 × 10^−2^ *vs.* 22.72 × 10^−2^ mPa·s), *B* (here: 654 *vs.* 684 K) and *T*_0_ (here: 172 *vs.* 160 K) are obtained. Taken all together, the ternary mixtures can be classified as fragile [39,44] in accordance with ionic liquid/Li-TFSA–based mixtures studied in literature [5,34,46].

**Figure 8:**
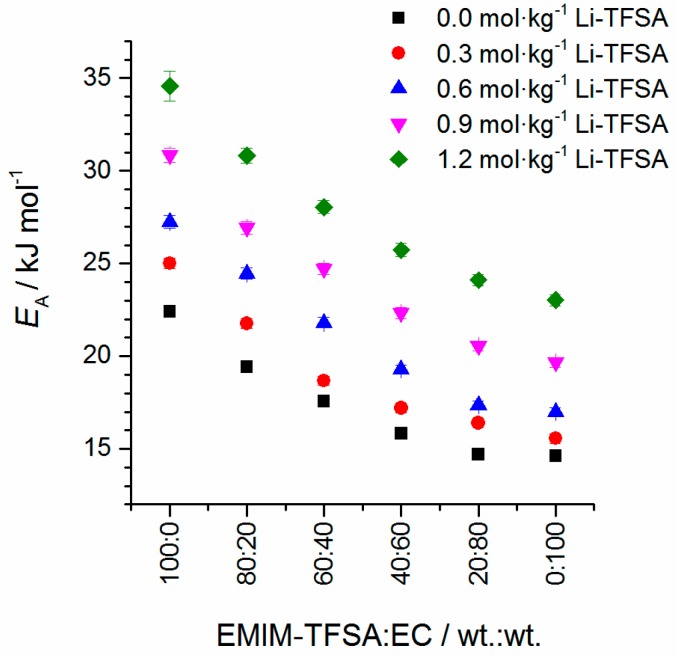
Activation energy of the flow process according to Arrhenius plotting in the temperature range of 50–100 °C (*Ṝ*^2^ = 0.991).

The well-known Arrhenius Equation (4) can be applied for calculating activation energies *E*_a_ of the flow process (*R* = universal gas constant; *η*_0_ = limiting viscosity). Nevertheless, it should be mentioned that an Arrhenius behavior of the temperature-dependent viscosity obviously cannot be observed (compare Figure 7). This is one of the results of the Angell strength parameter which controls how closely the electrolytes obey the Arrhenius law (ideal for *D* = ∞) [38]. Thus, it is important to know which temperature range is used for the fitting because the activating energy is highly dependent on the temperature range that is used. In this study, the temperature range of 50 to 100 °C is chosen for the fitting procedure and the results are depicted in Figure 8. The values are listed in the Supplementary Materials (Table S4).

(4) $$\eta =\;\eta_{0}\cdot \exp\left(\frac{E_{a}}{R\;\cdot \;T}\right)$$

The flow activation energy is influenced by the solvent composition as well as the Li-TFSA concentration. With the increase of the amount of EC in the mixture, the flow activation energy decreases steadily. At the same time, the addition of Li-TFSA increases the flow activation energy. The addition of Li-TFSA to an EMIM-TFSA/EC mixture causes the formation of highly solvated cluster-like structures where Li^+^ is solvated by TFSA^−^ ions or EC molecules, namely [Li(TFSA)_n_]^1−n^, [Li(EC)_m_]^+^ or [Li(EC)_n_(TFSA)_m_]^1−m^ complexes [22,23]. Thus, the intermolecular interactions increase significantly by applying Li-TFSA to a EC/EMIM-TFSA binary mixture [29,31]. Consequently, by adding Li-TFSA, the viscosity and flow activation energy of the mixture are enhanced and the mobility of the ions is reduced. These interactions are one factor which contributes to the viscosity value and flow characteristics. It should be noted that other factors such as ion pairing, intermolecular and inter-cluster interactions, molecular sizes, and ionic/molecular polarizability influence the flow characteristics greatly as well, and a differentiation between these parameters is exceedingly difficult. By increasing the EC content in the EMIM-TFSA/EC mixture, the viscosity values of the mixtures decrease significantly. This is due to the low viscosity value of <2 mPa·s of pure EC and weakened ionic aggregates [29].

**Figure 9:**
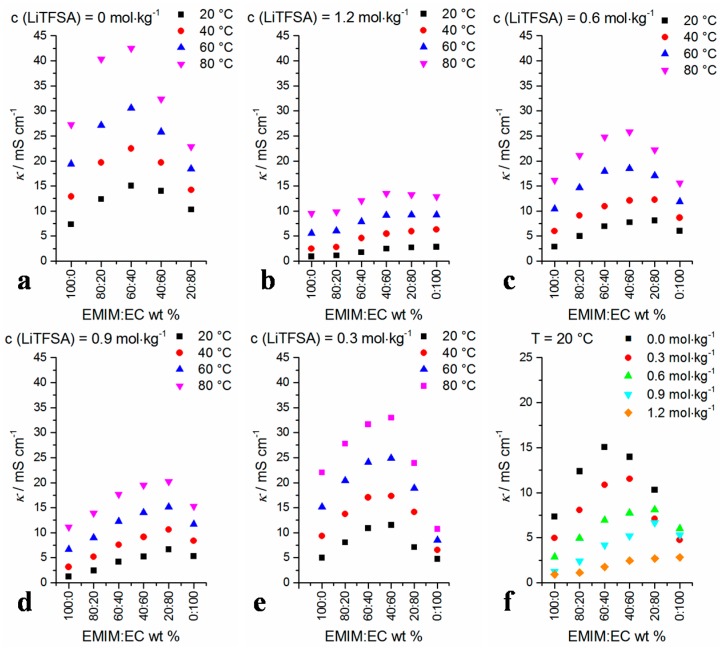
Temperature-dependent conductivity data of the mixtures *vs.* solvent ratio at selected Li-TFSA concentrations (**a**–**e**); In (**f**), the conductivity data at different Li-TFSA concentrations are compared at *T* = 20 °C.

**Figure 10:**
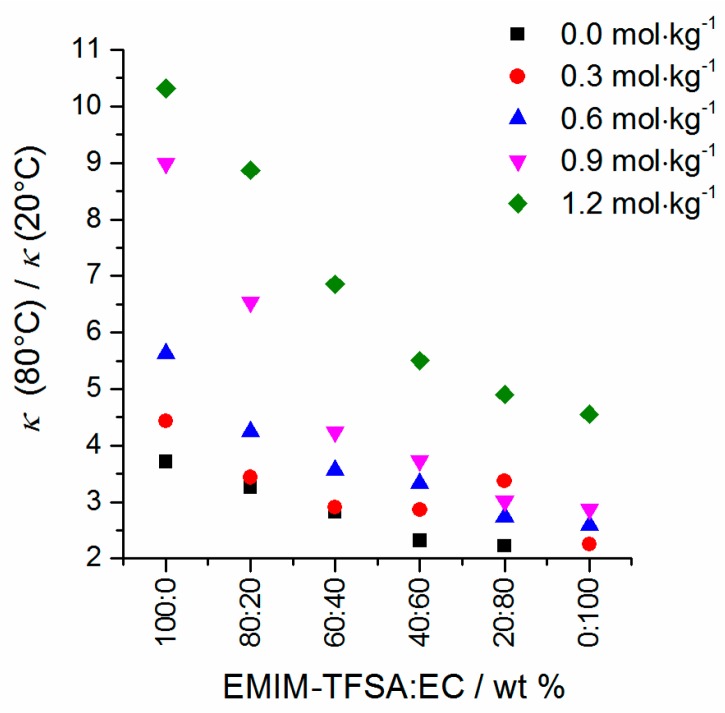
Temperature dependency of the conductivity data of the mixtures at selected EMIM-TFSA/EC mass fractions.

Temperature-dependent specific conductivity values of the mixtures are depicted in Figure 9. All data are listed in the Supplementary Materials (Table S5). As soon as Li-TFSA is added to an EMIM-TFSA/EC mixture, the conductivity values of the mixture usually decrease significantly (Figure 9f). This is in accordance with pyrrolidinium-TFSA/Li-TFSA mixtures in liquid state studied by Martinelli *et al.* [34] and IL/EC + Li-TFSA mixtures mentioned by Le *et al.* [47]. With increasing Li-TFSA content, the maximum value of the conductivity with respect to the EMIM-TFSA/EC ratio is measured in mixtures with higher EC amounts. Thus, the maximum shifts from EMIM-TFSA:EC ≈ 60:40 (without Li-TFSA) to EMIM-TFSA:EC ≈ 20:80 (*c*(Li-TFSA) = 1.2 mol·kg^−1^). As a consequence, the conductivity value order at different Li-TFSA concentrations is changed in the case of the mixture EMIM-TFSA:EC = 20:80 and the conductivity value of the mixture at *c*(Li-TFSA) = 0.3 mol·kg^−1^ (EMIM-TFSA:EC = 20:80) is lower than that of the mixture with *c*(Li-TFSA) = 0.6 mol·kg^−1^. At increased temperatures, the ion mobility is enhanced and the conductivity increases as well. The temperature dependency of the conductivity is quantified by calculating the quotient of absolute specific conductivity values at *T* = 80 °C and *T* = 20 °C (Figure 10) in terms of *κ*_80°C_/*κ*_20°C_.

It is conspicuous that the temperature dependency of the conductivity from the Li-TFSA concentration is at a maximum in pure EMIM-TFSA. However, a large jump can also be observed between *c*(Li-TFSA) = 0.6 mol·kg^−1^ and 1.2 mol·kg^−1^ in all solvent mass fractions. The temperature dependency of conductivity data and viscosity data follows the same trend which is an indication of a Walden-like behavior. Therefore, it is investigated if the Walden rule is also fulfilled for these ternary mixtures. Here, the limiting molar conductivity *Λ*_m_^0^ is replaced by the molar conductivity *Λ*_m_^cs+IL^, which consists of the amount of substance of the conducting salt (*c*s) *n*_cs_ and the contribution of the ionic liquid (IL) *n*_IL_ to the total molar conductivity. Thus, the ionic molar conductivity *Λ*_m_^cs+IL^ is composed of the temperature-dependent specific conductivity *κ*, the temperature-dependent density *d* of the mixture and the total mass *m* (Equation (5)).

(5) $$\Lambda_{m}^{cs+IL}=\;\frac{\kappa }{\left(\frac{m_{CS}}{M_{cs}}+\frac{m_{IL}}{M_{IL}}\right)\frac{d}{m}}$$

(6) $$\text{log }\Lambda_{m}^{cs+IL}=\log C^{\prime }+a\cdot \log\frac{1}{\eta }$$

It is found that the modified Walden rule can be applied even to the highest concentrated mixtures with *c*_cs+IL_ ≈ 4.9 mol∙dm^−3^. The fittings result in almost linear regressions according to Equation (6), with a mean coefficient of determination $\bar{R}$^2^ = 0.997 ± 0.002. The detailed results are listed in Table S6 in the Supplementary Materials. The results of the measurements and the behavior of an ideal classical dilute aqueous solution are plotted in Figure S3 (Supplementary Materials). It can be observed that small negative deviations from the ideal behavior are detected for ternary mixtures likewise. In the binary mixture EMIM-TFSA/Li-TFSA, the deviation from the classical ideal line is rather small, even at higher Li-TFSA concentrations. Such a result is indicative of the fact that similar contributions (equivalent impact) to viscosity and conductivity are caused in the binary EMIM-TFSA/Li-TFSA mixture and that each ion is almost equally influenced by all of its neighbors on the timescale of the measurement. Thus, isolated ion pairing between anions and cations does not occur, but rather a highly associated liquid mixture is formed [48]. Within a series of the same EMIM-TFSA/EC ratio but increasing Li-TFSA content, the negative deviation from the ideal line increases gradually. The largest negative deviation from the ideal classical behavior is received at the highest Li-TFSA concentrations (Figure S3) besides the increasing EC content [29]. Such a behavior indicates that stronger correlations influence either flow characteristics or the conductivity, which is in accordance with these measurements (Figure 7 and Figure 9f). Here, more isolated ion pairing and ionic cluster formation is supposed. In literature, the presence of such [Li(TFSA)_n_]_1−n_ and [Li(EC)_n_]^+^ clusters is studied by Raman and NMR spectroscopy extensively [22,23,49,50,51].

## 3. Materials and Methods

Ethylene carbonate (Sigma-Aldrich, Schnelldorf, Germany, 99%, anhydrous), 1-ethyl-3-methylimidazolium bis(trifluoromethanesulfonyl)azanide (EMIM-TFSA, Iolitec, Heilbronn, Germany, 99%) and lithium bis(trifluoromethanesulfonyl)azanide (Li-TFSA, Iolitec) were dried before usage. For this purpose, the reagents were placed into a Thermoprep oven (831 KF Coulometer and 860 KF Thermoprep oven from Metrohm, Filderstadt, Germany, 120 °C). A continuous gas flow (dried air) was bubbled through the liquid until a water drift of <1 µg·min^−1^ was detected by the Coulometer (Metrohm, Filderstadt, Germany). After this drying procedure, the water content of the solvents was determined to be less than 20 ppm. The preparation of the mixtures was performed in an argon-filled glove box (MBraun GmbH, Garching, Germany) with oxygen and water levels below 0.5 ppm. All samples were weighted inside the glovebox (Sartorius, AX 224, Göttingen, Germany) where a standard uncertainty *u* of *u*(*χ*) = 0.0002 was achieved. Additionally, all measurement were received at atmospheric pressure (*p*) and are reported to 0.1 MPa (detected by using a mercury barometer) with standard uncertainty (*u*) *u*(*p*) = 5 kPa.

The measurement of the viscosity, conductivity, density and differential scanning calorimetry (DSC) was performed as described elsewhere in detail [29]. Briefly, the viscosity was studied using a rotational rheometer (cone/plate geometry, Malvern Gemini HR Nano, Worcestershire, UK) in range of *T* = (20–120) °C (shear rate of 100 s^−1^). For measuring the conductivity, a device from RHD instruments (closed cell, Marburg, Germany) was used and the measurement was done by the standard complex impedance method (Zahner Zennium IM6 electrochemical workstation, Kronach, Germany; frequency range: 1 kHz to 1 MHz; ac-offset: 10 mV; the cell constant *C* was received by measuring a standard solution of 1.413 mS·cm^−1^ at 25 °C, Hanna Instruments, HI 70031; *u*(*C*) = 0.01 *C*). The density values were obtained by a precision densitometer from Anton Paar (DMA 4500 M, Graz, Austria) in a temperature range between *T* = (20–80) °C. The standard uncertainty of the temperature during the measurement was *u*(*T*) = 0.01 °C. The mixtures were investigated by DSC with respect to the phase behavior (Netzsch DSC 204 F1 Phoenix^®^, Selb, Germany; Al crucibles closed; heating and cooling rates are specified in the manuscript text). Standard uncertainty *u* of the temperature is *u*(*T*) = 3 K.

## 4. Conclusions

The ternary mixtures composed of EMIM-TFSA, EC and Li-TFSA are described in this study based on temperature-dependent data of conductivity, viscosity and density at *T* = (20–80) °C. DSC measurements are performed for evaluating the phase behavior of the mixtures within *T* = (−150–100) °C to evaluate phase transitions and glass temperatures. It could be demonstrated that the mixtures can be classified as fragile according to the fragility concept. It is shown that the addition of Li-TFSA influences both solvents strongly and affects the properties of the mixtures significantly. It is proved that the Walden rule can be applied even to highly concentrated ternary mixtures. The data can be used as experimental proof for theoretical calculations of molecular structures and ionic clusters in more complex ternary mixtures and are supposed to be a helpful data set for applications in electrochemical and physicochemical approaches.

## Figures and Tables

**Table 1:** Overview of the mixtures and selected properties (density (*d*), conductivity (*κ*), viscosity (*η*), melting temperature (*T*_m_), glass transition temperature (*T*_g_)). Data of the solvent mixtures at pressure *p* = 0.1 MPa. Standard uncertainties *u* are *u*(*d*) = 0.0005 g·cm^−3^; *u*(*p*) = 5 kPa; *u*(*η*) = 0.05·*η*; *u*(*κ*) = 0.03 *κ*; *u*(*T*, viscosity) = 0.1 K; *u*(*T*, density) = 0.01 K; *u*(*T*; DSC) = 3 °C; *u*(*T*, conductivity) = 0.1 K.

EMIM-TFSA:EC (wt/wt)	*c*/mol·kg^−1^ Li-TFSA	*d*/g·cm^−3^ (20 °C)	*κ*/mS·cm^−1^ (20 °C)	*η*/mPa·s (20 °C)	*T*_m_^1^/°C (peak max)	*T*_g_^1^/°C ^4^
100:0	0	1.5208	7.34	41.3	−4.0	−85.3
100:0	0.3	–	4.97	70.2	−19.6	−83.6
100:0	0.6	1.5848	2.87	123.4	– ^2^	−73.9
100:0	0.9	–	1.24	288.0	– ^2^	−66.1
100:0	1.2	1.6276	0.92	555.9	– ^2^	−63.9
80:20	0	1.4858	12.36	15.3	−14.1	−91.9
80:20	0.3	–	8.08	27.1	– ^2^	−85.9
80:20	0.6	1.5480	4.96	51.1	– ^2^	−79.5
80:20	0.9	–	2.43	105.0	– ^2^	−72.5
80:20	1.2	1.6161	1.11	230.0	– ^2^	−66.1
60:40	0	1.4476	15.68	7.9	9.3	−96.6
60:40	0.3	–	10.88	11.6	−0.7	−89.6
60:40	0.6	1.5122	6.94	24.0	– ^2^	−83.2
60:40	0.9	–	4.17	50.1	– ^2^	−75.6
60:40	1.2	1.5933	1.76	110.0	– ^2^	−67.7
40:60	0	1.4096	14.42	4.8	23.6	– ^2^
40:60	0.3	–	11.53	7.9	15.0	−90.2
40:60	0.6	1.4868	7.75	13.4	5.5	−85.5
40:60	0.9	–	5.22	28.2	−5.9	−78.6
40:60	1.2	1.5656	2.46	65.3	– ^2^	−68.5
20:80	0	1.3765	10.30	3.5	34.5	– ^2^
20:80	0.3	–	7.09	5.7	28.9	−70.7
20:80	0.6	1.4554	8.11	9.72	20.5	−67.6
20:80	0.9	–	6.68	18.0	15.3	−82.8
20:80	1.2	1.5353	2.70	41.0	−5.3	−81.0
0:100	0	1.3219 ^3^	–	–	41.6	– ^2^
0:100	0.3	–	4.76	4.2	37	−55.5
0:100	0.6	1.4321	6.02	6.7	28.8	−60.3
0:100	0.9	–	5.31	12.9	20.3	−57.9
0:100	1.2	1.5139	2.83	26.5	7.5	−72.4

^1^ DSC: heating at 10 K∙min^−1^; ^2^ No melting point or glass transition could be extracted from the measurement; ^3^ *T* = 40 °C; ^4^ the glass transition temperature *T*_g_ was received at the point of inflection.

## Supplementary Materials

Supplementary materials can be found at http://www.mdpi.com/1422-0067/17/5/670/s1.

## Abbreviations

EC: Ethylene carbonate
EMIM-TFSA: 1-Ethyl-3-methylimidazolium bis(trifluoromethanesulfonyl)azanide
Li-TFSA: Lithium bis(trifluoromethanesulfonyl)azanide
DSC: Differential scanning calorimetry
VFTH: Vogel-Fulcher-Tammann-Hesse
