# Supplementary material for: Investigation of Ternary Mixtures Containing 1-Ethyl-3-methylimidazolium Bis(trifluoromethanesulfonyl)azanide, Ethylene Carbonate and Lithium Bis(trifluoromethanesulfonyl)azanide
Source: Int J Mol Sci. 2016 May 4;17(5):670. doi: 10.3390/ijms17050670 (PMC4881496; doi:10.3390/ijms17050670)
Supplement: Supplementary file 1 [file ijms-17-00670-s001.pdf]

Supplementary Materials: Investigation of Ternary Mixtures Containing 1-Ethyl-3-methylimidazolium Bis(trifluoromethanesulfonyl)-azanide, Ethylene Carbonate and Lithium Bis(trifluoromethanesulfonyl)azanide

Andreas Hofmann, Matthias Migeot, Lukas Arens and Thomas Hanemann

Table S1. Density data of the mixtures with Li-TFSA at pressure p = 0.1 MPa (Standard uncertainties u are $u(d)$ = 0.0005 g·cm^{-3}, $u(p)$ = 5 kPa, $u(T)$ = 0.01 K).

EMIM-TFSA:EC (wt/wt)	Li-TFSA mol·kg^{-1}	d g·cm^{-3} 20 °C	d g·cm^{-3} 40 °C	d g·cm^{-3} 60 °C	d g·cm^{-3} 80 °C
100:0	0	1.5240	1.5038	1.4838	1.4642
80:20	0	1.4858	1.4646	1.4438	1.4232
60:40	0	1.4476	1.4259	1.4044	1.3831
40:60	0	1.4096	1.3873	1.3653	1.3434
20:80	0	1.3765	1.3541	1.3316	1.3092
0:100	0	–	1.3219	1.2991	1.2763
100:0	0.6	1.5848	1.5637	1.5428	1.5222
80:20	0.6	1.5480	1.5265	1.5051	1.484
60:40	0.6	1.5122	1.4903	1.4684	1.4466
40:60	0.6	1.4868	1.4643	1.4422	1.4202
20:80	0.6	1.4554	1.4326	1.4101	1.3877
0:100	0.6	1.4321	1.4032	1.3816	1.3591
100:0	1.2	1.6276	1.6055	1.5837	1.5619
80:20	1.2	1.6161	1.5939	1.5720	1.5502
60:40	1.2	1.5933	1.5707	1.5485	1.5266
40:60	1.2	1.5656	1.5428	1.5203	1.4980
20:80	1.2	1.5353	1.5126	1.4899	1.4670
0:100	1.2	1.5139	1.4907	1.4677	1.4449

Table S2a. Linear fitting ($ax + b$) of density data according to EC mass percentage (density *vs.* EC mass percentage at specified Li-TFSA concentration).

Li-TFSA	T °C	a 10^{-3} g·cm^{-3}	b g·cm^{-3}	R^2
0	20	−1.86 ± 0.03	1.5229	0.999
0	40	−1.83 ± 0.04	1.5010	0.998
0	60	−1.86 ± 0.04	1.4808	0.998
0	80	−1.88 ± 0.04	1.4610	0.997
0.6	20	−1.52 ± 0.06	1.5794	0.992
0.6	40	−1.59 ± 0.05	1.5594	0.996
0.6	60	−1.60 ± 0.05	1.5382	0.995
0.6	80	−1.62 ± 0.05	1.5174	0.995
1.2	20	−1.20 ± 0.07	1.6436	0.983
1.2	40	−1.21 ± 0.07	1.6215	0.983
1.2	60	−1.22 ± 0.07	1.5998	0.983
1.2	80	−1.23 ± 0.07	1.5784	0.983

The average of the slope $\overline{m}$ at 0 mol·kg^{-1}, 0.6 mol·kg^{-1} and 1.2 mol·kg^{-1} is calculated as $\overline{m} = \frac{1}{n}\sum_{i=1}^{n} m_i$. The error Δ of the average value is determined by $\Delta = \frac{1}{n}\sqrt{\sum_{i=1}^{n}\Delta_i^2}$.

Table S2b. Second order of polynomial fitting ($ax^2 + bx + c$) of density data according to EC mass percentage (density *vs.* EC mass percentage at specified Li-TFSA concentration).

Li-TFSA	*T* °C	*a* 10^{-6} g·cm^{-3}	*b* 10^{-3} g·cm^{-3}	*c* g·cm^{-3}	R^2
0	20	1.86 ± 0.77	−2.00 ± 0.06	1.5229	0.9996
0	40	2.56 ± 0.43	−2.08 ± 0.04	1.5010	0.9998
0	60	2.71 ± 0.42	−2.13 ± 0.04	1.4808	0.9998
0	80	2.87 ± 0.42	−2.17 ± 0.04	1.4610	0.9998
0.6	20	3.79 ± 0.89	−1.90 ± 0.09	1.5794	0.9986
0.6	40	2.54 ± 1.04	−1.84 ± 0.11	1.5594	0.9982
0.6	60	2.88 ± 1.00	−1.88 ± 0.10	1.5382	0.9983
0.6	80	3.03 ± 1.02	−1.92 ± 0.11	1.5174	0.9983
1.2	20	−3.55 ± 1.92	−0.84 ± 0.20	1.6436	0.9892
1.2	40	−3.57 ± 1.88	−0.85 ± 0.20	1.6215	0.9897
1.2	60	−3.59 ± 1.91	−0.86 ± 0.20	1.5998	0.9897
1.2	80	−3.65 ± 1.98	−0.87 ± 0.21	1.5784	0.9891

Table S3. Free VFTH fitting of the viscosity data. For the fitting procedure, the following initial fitting parameters are used: η_0 = 0.2 mPa·s; D = 4; T_0 = 160 K. No additional assumptions were done during the fitting procedure otherwise mentioned (Standard uncertainties u are $u(T_g)$ = 3 °C, $u(D) = 0.025 \cdot D$, $u(m) = 0.03 \cdot m$, $u(T_0) = 0.015 \cdot T_0$, $u(\eta_0) = 0.05 \cdot \eta_0$).

Ratio EMIM-TFSA:EC (wt/wt)	*c*(Li-TFSA) mol·kg^{-1}	η_0 10^{-2} mPa·s	T_0/K	D ± (≤0.1)	m	T_g/K DSC *	R^2	$\eta_{20°C}/\eta_{120°C}$
100:0	0	18.3 ± 0.9	172.2 ± 1.5	3.8	171 ± 4	187.85	0.9997	11.3
100:0	0.3	19.9 ± 0.8	181.1 ± 1.1	3.6	178 ± 4	189.55	0.9998	16.0
100:0	0.6	26.3 ± 0.9	195.4 ± 0.7	3.1	207 ± 3	199.25	0.9999	22.2
100:0	0.9	29.3 ± 1.2	200.9 ± 0.7	3.1	204 ± 3	207.05	0.9999	34.3
100:0	1.2	31.1 ± 2.7	190.0 ± 1.4 ***	4.1	160 ± 5	209.25	0.9998	45.9
80:20	0	11.7 ± 0.8	158.2 ± 2.4	4.2	157 ± 7	181.25	0.9994	7.8
80:20	0.3	18.8 ± 0.7	183.8 ± 1.2	3.0	215 ± 5	187.25	0.9998	10.8
80:20	0.6	21.7 ± 0.8	191.8 ± 0.7	2.9	220 ± 4	193.65	0.9998	15.2
80:20	0.9	25.0 ± 0.9	197.9 ± 0.7	2.9	220 ± 4	200.65	0.9998	21.1
80:20	1.2	27.6 ± 1.2	205.7 ± 0.7	2.8	223 ± 4	207.05	0.9998	34.7
60:40	0	6.6 ± 0.1	132.9 ± 0.4	5.8	118 ± 1	176.55	0.9999	6.1
60:40	0.3	16.8 ± 0.1	191.2 ± 0.3	2.7	238 ± 2	183.55	0.9998	7.8
60:40	0.6	17.1 ± 0.2	184.7 ± 0.3	2.9	218 ± 1	189.95	0.9998	10.7
60:40	0.9	19.9 ± 0.2	194.1 ± 0.2	2.8	225 ± 1	197.55	0.9999	16.2
60:40	1.2	24.3 ± 0.2	203.3 ± 0.1	2.7	234 ± 1	205.45	0.9999	24.4
40:60	0	6.0 ± 0.1	129.3 ± 0.5	5.6	121 ± 1	-- **	0.9999	5.0
40:60	0.3	13.6 ± 0.1	170.4 ± 0.5	2.9	216 ± 2	182.95	0.9998	6.2
40:60	0.6	16.6 ± 0.2	181.8 ± 0.4	2.7	235 ± 2	187.65	0.9997	7.9
40:60	0.9	18.8 ± 0.2	191.3 ± 0.3	2.7	237 ± 2	194.55	0.9998	11.3
40:60	1.2	21.8 ± 0.2	199.5 ± 0.2	2.6	237 ± 1	204.65	0.9999	18.3
20:80	0	6.3 ± 0.1	130.8 ± 0.5	5.0	134 ± 1	-- **	0.9998	6.0
20:80	0.3	11.2 ± 0.1	162.5 ± 0.6	3.2	202 ± 2	202.45	0.9997	5.6
20:80	0.6	20.3 ± 0.2	185.3 ± 0.5	2.3	277 ± 3	205.55	0.9995	6.3
20:80	0.9	19.0 ± 0.2	189.7 ± 0.4	2.4	253 ± 2	190.35	0.9997	9.5
20:80	1.2	22.0 ± 0.2	199.1 ± 0.3	2.4	255 ± 1	192.15	0.9998	14.3
0:100	0	3.1 ± 0.1	109.9 ± 0.2	7.1	99 ± 1	187.95	0.9999	4.0
0:100	0.3	7.9 ± 0.1	145.3 ± 0.6	4.0	162 ± 2	217.65	0.9997	4.7
0:100	0.6	11.7 ± 0.1	166.8 ± 0.6	3.1	207 ± 2	212.85	0.9996	5.9
0:100	0.9	15.5 ± 0.2	186.1 ± 0.5	2.5	249 ± 2	215.25	0.9996	8.5
0:100	1.2	16.7 ± 0.2	192.6 ± 0.3	2.6	238 ± 2	200.75	0.9997	12.4

* DSC: heating at 10 K/min; ** No T_g could be extracted from the measurement; *** the lower fitting border of T_0 was set to 190 K.

For the fitting procedure, the following initial fitting parameters are used: η_0 = 0.2 mPa·s; D = 4; T_0 = 160 K. No additional assumptions were done during the fitting procedure otherwise mentioned.

Table S4. Data of flow activation energies.

EMIM-TFSA:EC (wt/wt)	Li-TFSA mol·kg^{-1}	E_A kJ·mol^{-1}	R^2
100:0	0	22.4 ± 0.2	0.993
80:20	0	19.4 ± 0.2	0.991
60:40	0	17.6 ± 0.2	0.990
40:60	0	15.8 ± 0.2	0.989
20:80	0	14.7 ± 0.2	0.986
0:100	0	14.6 ± 0.3	0.992
100:0	0.3	25.0 ± 0.3	0.993
80:20	0.3	21.8 ± 0.3	0.993
60:40	0.3	18.7 ± 0.2	0.992
40:60	0.3	17.2 ± 0.2	0.989
20:80	0.3	16.4 ± .02	0.989
0:100	0.3	15.6 ± 0.3	0.985
100:0	0.6	27.3 ± 0.3	0.992
80:20	0.6	24.2 ± 0.3	0.993
60:40	0.6	21.8 ± 0.3	0.991
40:60	0.6	19.3 ± 0.2	0.992
20:80	0.6	17.4 ± 0.2	0.990
0:100	0.6	17.0 ± 0.2	0.989
100:0	0.9	30.9 ± 0.4	0.992
80:20	0.9	26.9 ± 0.3	0.991
60:40	0.9	24.7 ± 0.3	0.992
40:60	0.9	22.3 ± 0.3	0.991
20:80	0.9	20.6 ± 0.3	0.992
0:100	0.9	19.7 ± 0.2	0.992
100:0	1.2	34.6 ± 0.8	0.992
80:20	1.2	30.8 ± 0.4	0.991
60:40	1.2	28.1 ± 0.3	0.992
40:60	1.2	25.7 ± 0.4	0.990
20:80	1.2	24.1 ± 0.3	0.992
0:100	1.2	23.0 ± 0.3	0.991

Table S5. Conductivity data of the solvent mixtures at pressure p = 0.1 MPa (Standard uncertainties u are $u(\chi)$ = 0.0002, $u(\kappa)$ = 0.03·κ, $u(p)$ = 5 kPa, $u(\chi)$ = 0.0002, $u(T)$ = 0.1 K).

EMIM-TFSA:EC (wt/wt)	χ EC	c(Li-TFSA) mol·kg^{-1}	κ mS·cm^{-1} 20 °C $u(\kappa)$ = 0.03·κ	κ mS·cm^{-1} 40 °C $u(\kappa)$ = 0.04·κ	κ mS·cm^{-1} 60 °C $u(\kappa)$ = 0.05·κ	κ mS·cm^{-1} 80 °C $u(\kappa)$ = 0.05·κ
100:0	0	0	7.34	13.48	20.56	28.93
80:20	0.526	0	12.37	19.69	27.14	40.37
60:40	0.748	0	15.68	22.47	30.53	42.53
40:60	0.870	0	14.42	20.45	26.95	35.92
20:80	0.912	0	10.3	14.23	18.42	22.89
0:100	1.000	0	--	--	--	--
100:0	0	0.3	4.97	9.33	15.16	22.03
80:20	0.526	0.3	8.08	13.73	20.43	27.78
60:40	0.748	0.3	10.88	17.06	24.12	31.67
40:60	0.870	0.3	11.53	17.35	24.9	32.99
20:80	0.912	0.3	7.09	14.15	18.89	23.92
0:100	1.000	0.3	4.76	6.53	8.54	10.73
100:0	0	0.6	2.87	6.01	10.42	16.13
80:20	0.526	0.6	4.96	9.09	14.67	21.09
60:40	0.748	0.6	6.94	10.92	17.96	24.76
40:60	0.870	0.6	7.75	12.1	18.5	25.8
20:80	0.912	0.6	8.11	12.27	17.02	22.23
0:100	1.000	0.6	6.02	8.66	11.9	15.59
100:0	0	0.9	1.24	3.15	6.7	11.12
80:20	0.526	0.9	2.43	5.18	9	13.9
60:40	0.748	0.9	4.17	7.56	12.24	17.69
40:60	0.870	0.9	5.22	9.14	14.01	19.52
20:80	0.912	0.9	6.68	10.59	15.12	20.2
0:100	1.000	0.9	5.31	8.39	11.73	15.29
100:0	0	1.2	0.92	2.46	5.58	9.49
80:20	0.526	1.2	1.11	2.8	6.05	9.82
60:40	0.748	1.2	1.76	4.59	7.86	12.06
40:60	0.870	1.2	2.46	5.48	9.13	13.56
20:80	0.912	1.2	2.70	5.93	9.24	13.26
0:100	1.000	1.2	2.83	6.29	9.19	12.9

Table S6. Results of Walden plots and linear regression.

Ratio (wt/wt) EMIM-TFSA:EC	*c*(Li-TFSA) mol·kg^{-1}	Slope (*a*)	R^2
100:0	0	0.75 ± 0.01	0.999
100:0	0.3	0.75 ± 0.02	0.998
100:0	0.6	0.75 ± 0.02	0.998
100:0	0.9	0.83 ± 0.03	0.997
100:0	1.2	0.83 ± 0.03	0.997
80:20	0	0.79 ± 0.03	0.997
80:20	0.3	0.72 ± 0.01	0.999
80:20	0.6	0.73 ± 0.02	0.997
80:20	0.9	0.77 ± 0.02	0.999
80:20	1.2	0.82 ± 0.03	0.995
60:40	0	0.80 ± 0.01	0.999
60:40	0.3	0.74 ± 0.02	0.999
60:40	0.6	0.73 ± 0.02	0.998
60:40	0.9	0.71 ± 0.02	0.998
60:40	1.2	0.80 ± 0.03	0.996
40:60	0	0.73 ± 0.04	0.992
40:60	0.3	0.83 ± 0.03	0.997
40:60	0.6	0.81 ± 0.04	0.993
40:60	0.9	0.73 ± 0.02	0.998
40:60	1.2	0.78 ± 0.02	0.998
20:80	0	0.79 ± 0.01	0.999
20:80	0.3	0.89 ± 0.05	0.991
20:80	0.6	0.76 ± 0.02	0.999
20:80	0.9	0.68 ± 0.01	0.999
20:80	1.2	0.79 ± 0.03	0.995
0:100	0	–	–
0:100	0.3	0.73 ± 0.02	0.998
0:100	0.6	0.75 ± 0.02	0.998
0:100	0.9	0.70 ± 0.01	0.999
0:100	1.2	0.69 ± 0.04	0.998

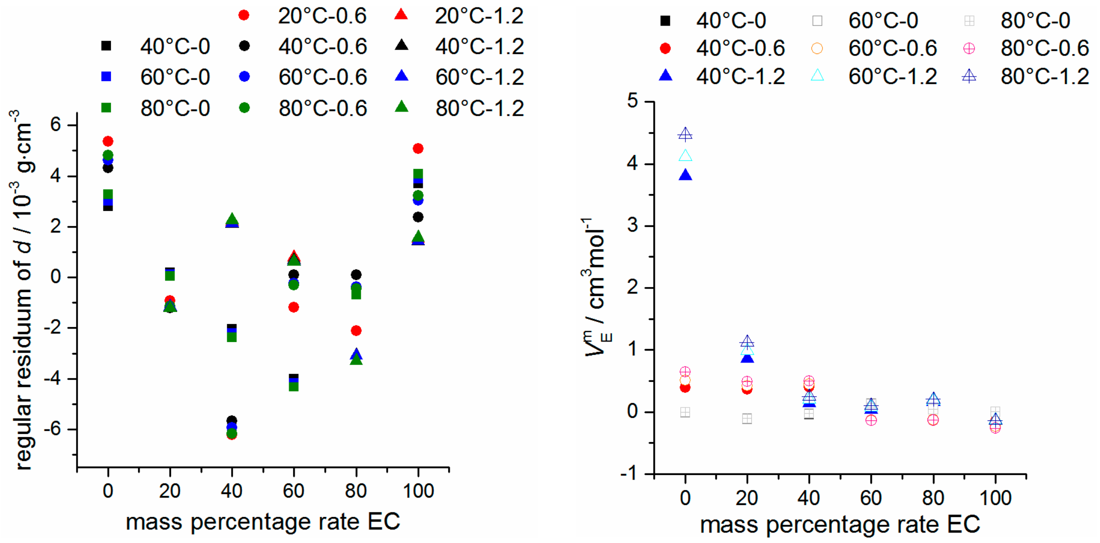

(**a**) (**b**)

Figure S1. Residuum (**a**) of the real density of selected mixtures from the linear ideal behavior; Molar excess volume (**b**) of EMIM-TFSA/EC/Li-TFSA ternary mixtures as a function of the EC mass percentage rate (related to the EMIM-TFSA/EC solvent mixture) at different temperatures.

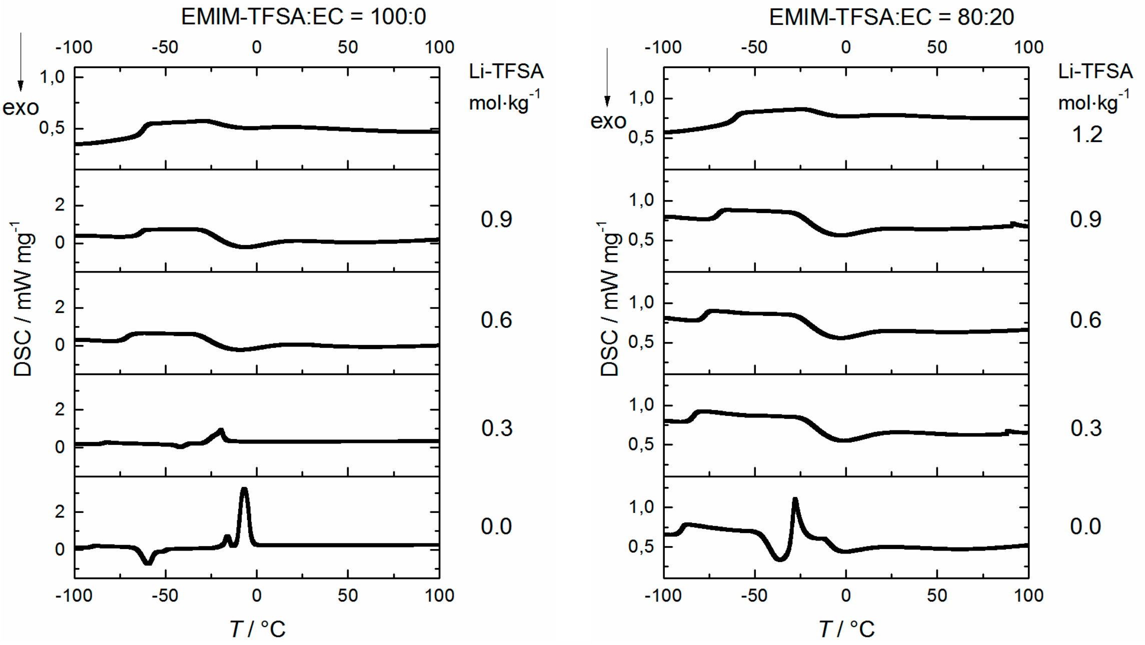

(**a**) (**b**)

Figure S2. *Cont.*

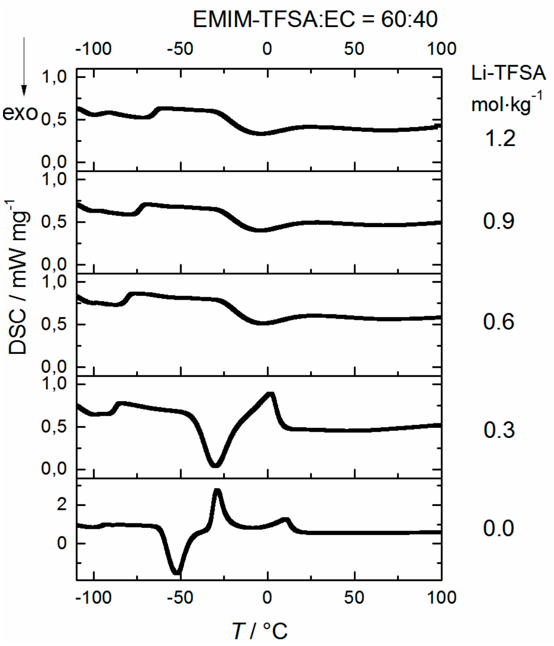

(**c**)

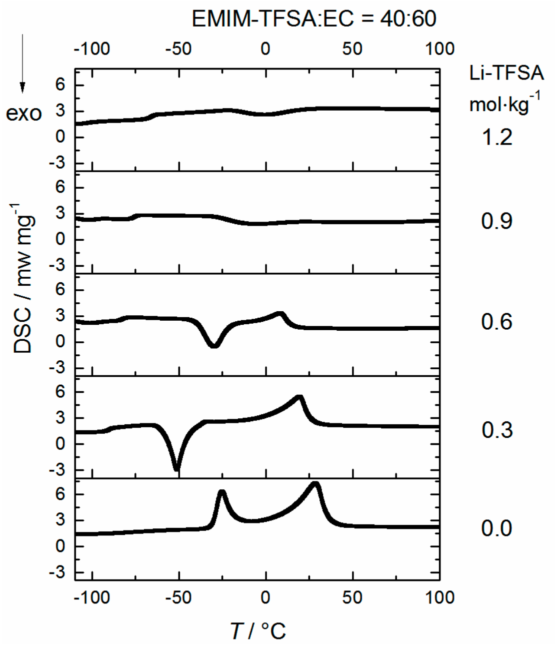

(**d**)

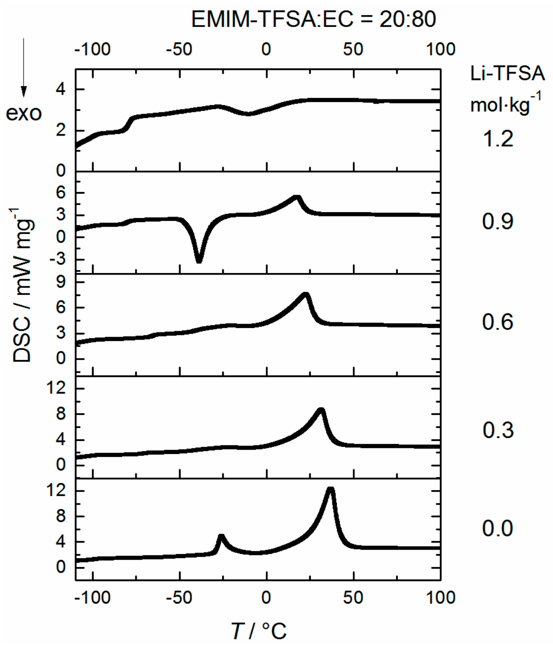

(**e**)

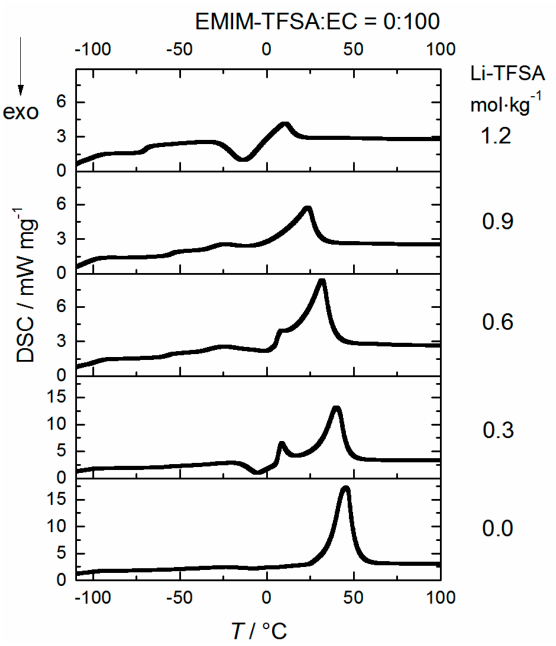

(**f**)

Figure S2. (**a**–**f**) DSC measurements during heating (20 K·min^{-1}) in the temperature range of −110 to 100 °C (exo down) of EMIM-TFSA: EC (wt/wt) + Li-TFSA mixtures. The concentration of Li-TFSA is mentioned on the right hand side of each figure. The composition of the solvent (EMIM-TFSA:EC) is written on the top of each figure. The figures are arranged in a descending order with respect to EC concentration.

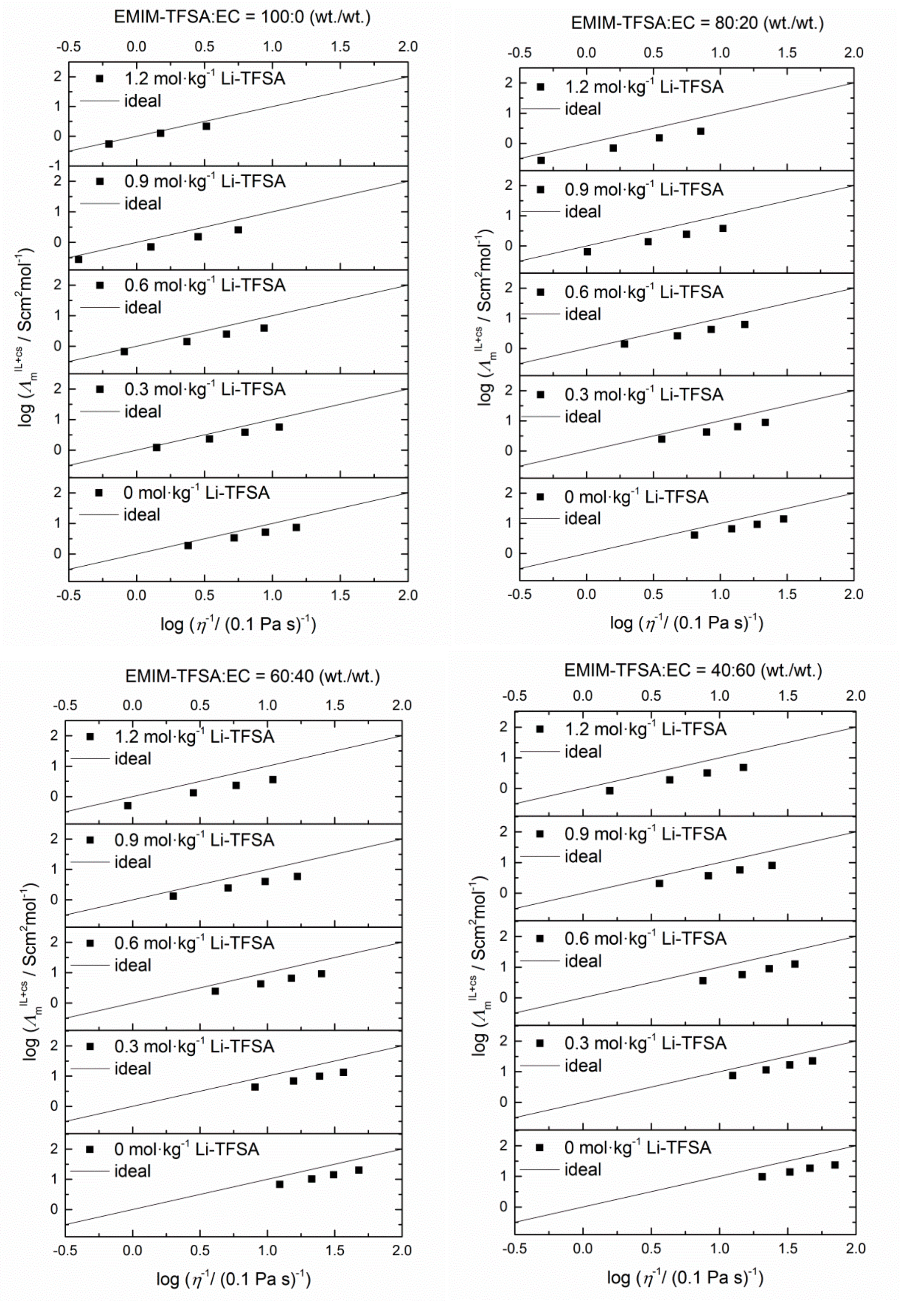

Figure S3. *Cont.*

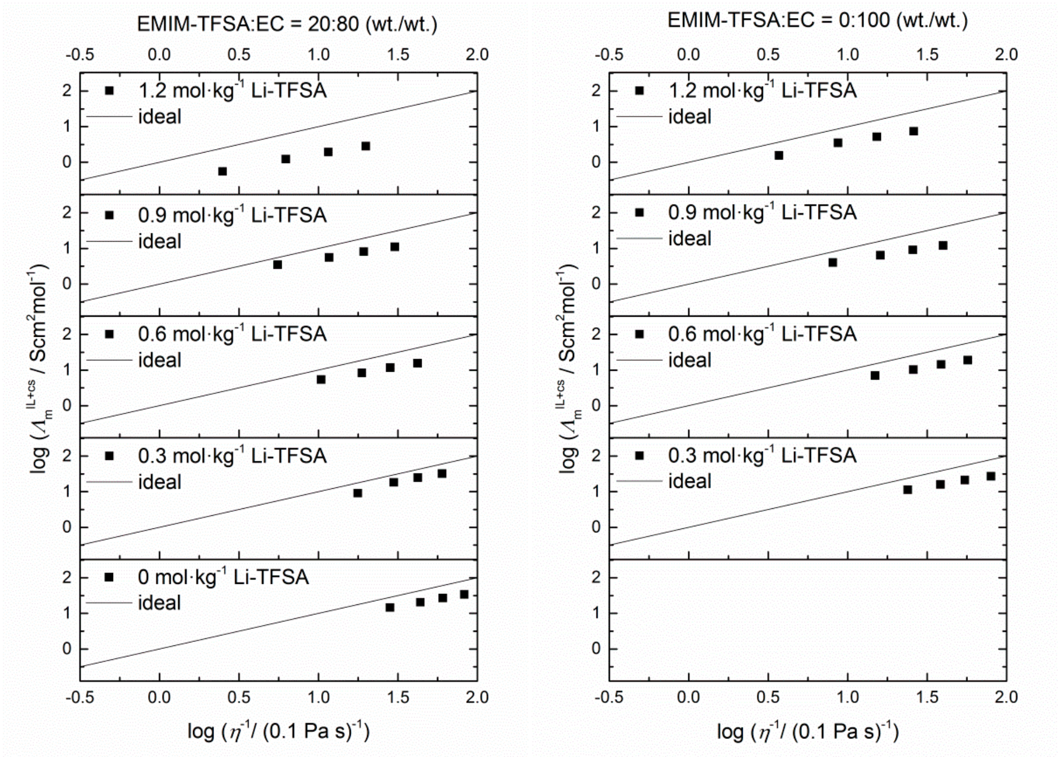

Figure S3. Walden plots of temperature-dependent (20–80°C) viscosity and conductivity data of the mixtures (EMIM-TFSA:EC = wt/wt) with selected Li-TFSA concentrations. The results of the measurements are depicted as black squares, whereas the black line displays the behavior of an ideal classical dilute aqueous solution (slope of one, labelled as "fit"). For a better comparison with plots in literature, the viscosity value is shown in units of Poise (0.1 Pa·s).
